# Exploring Factors and Associate Responses for Anxiety in the Coronavirus Disease 2019 Pandemic: A Web-Based Survey in Japan

**DOI:** 10.3389/fpsyg.2021.795219

**Published:** 2022-03-08

**Authors:** Jinman Zhuang, Guoxi Cai, Yixiao Lu, Xinying Xu, Yulan Lin, Li Ping Wong, Zhijian Hu, Taro Yamamoto, Kouichi Morita, Kiyoshi Aoyagi, Fei He

**Affiliations:** ^1^Fujian Provincial Key Laboratory of Environment Factors and Cancer, Department of Epidemiology and Health Statistics, School of Public Health, Fujian Medical University, Fuzhou, China; ^2^Department of Public Health, Nagasaki Prefectural Institute of Environment and Public Health, Nagasaki, Japan; ^3^Department of International Health, Institute of Tropical Medicine (NEKKEN), Nagasaki University, Nagasaki, Japan; ^4^Department of Public Health, Nagasaki University Graduate School of Biomedical Sciences, Nagasaki, Japan; ^5^Centre for Epidemiology and Evidence-Based Practice, Department of Social and Preventive Medicine, Faculty of Medicine, Universiti Malaya, Kuala Lumpur, Malaysia; ^6^Department of Virology, Institute of Tropical Medicine (NEKKEN), Nagasaki University, Nagasaki, Japan

**Keywords:** anxiety, COVID-19, STAI-6, mental health, associate response

## Abstract

**Background and Objectives:**

Anxiety plays an important role in psychology. An exploration of anxiety and its associated reactions may provide insight into measures for addressing mental health problems caused by the coronavirus disease 2019 (COVID-19) pandemic. Data from this study provide potential correlational responses to anxiety.

**Methods:**

A cross-sectional study using data collected *via* an online self-reported questionnaire was conducted in Japan during the COVID-19 pandemic. Using the State-Trait Anxiety Inventory (STAI-6), we assessed anxiety levels and explored the relationship between anxiety STAI-6 score, sources of COVID-19 information, the influences of COVID-19, social anxiety symptoms, discrimination, and evaluation of the government.

**Results:**

A total of 4,127 participants were included in the analysis. The level of anxiety was not equally distributed across the general population with respect to age, gender, educational level, occupation, income, presence of underlying disease, and location (*p* < 0.05). The number of sources of information on COVID-19 had a positive correlation with the STAI-6 score (Spearman’s rho = 0.176, *p* < 0.001). The influence of the pandemic was correlated with moderate–severe anxiety. A high level of anxiety added to social anxiety (Spearman’s rho = 0.04, *p* < 0.05) and discriminatory behavior (Spearman’s rho = 0.11, *p* < 0.01). Being female (Estimate = 0.926) and from a non-emergency area (Estimate = −0.308) was related to higher STAI scores, and higher education (Estimate = −0.305), and income (Estimate = −0.168) decreased the STAI score. The respondents who had a lower evaluative score of the preventive activities undertaken by the national and local governments had higher STAI-6 scores.

**Conclusion:**

Our findings provide statistical evidence for the associated reaction of anxiety and that anxiety reactions may vary in predictable ways. Further studies should focus on the strategic interventions that may decrease the associated responses to anxiety, to address mental health issues due to the pandemic.

## Introduction

The role of psychology in healthcare has significantly expanded in recent years. Anxiety level is a key indicator of mental health (Horenstein and Heimberg, 2020); persistent moderate to severe anxiety symptoms can have a negative impact on psychological health (Labrague and De Los Santos, 2020).

Due to the coronavirus disease 2019 (COVID-19) pandemic, dramatic changes to normal life emerged in many countries. The general population experienced psychological distress, especially anxiety, during the pandemic (Liu X. et al., 2020; Wang et al., 2020). Individuals with high-level anxiety were found to be vulnerable to changes in harmless bodily sensations as they are affected by the circumstances of COVID-19 (Asmundson and Taylor, 2020). A recently published manuscript in Nature showed that anxiety among graduate students in the United States rose by 50% during the pandemic compared with the previous year (Woolston, 2020). Therefore, anxiety is an important concern during an epidemic.

Excessive online searching for health-related information was found to be anxiety-provoking (McMullan et al., 2019). Furthermore, different information sources related to the COVID-19 pandemic contributed differently to responses to the outbreak (Cui et al., 2020). Also, anxiety itself was a reason for excessive searching for pandemic-related information (Vai et al., 2020). Importantly, social anxiety is marked by fear or anxiety of possible scrutiny by others in social situations (Heimberg et al., 2014). Therefore, the accumulation of individual anxiety may trigger a series of social anxiety symptoms (Ng-Cordell et al., 2018). It was noted that trust is beneficial to reducing anxiety (Flugelman et al., 2020; Jhang, 2020); in other words, anxiety may add distrust regarding relevant departments. A previous study showed that a lower level of anxiety was associated with trust in the healthcare system (AlRuthia et al., 2020). Further, a high level of anxiety may contribute to the increased possibility of being affected by a public health emergency (Tull et al., 2020) and the influences of a pandemic may in turn increase the anxiety. Thus, there may be certain unidentified factors that add to anxiety and cause a ripple effect.

The COVID-19 outbreak has spread to countries worldwide in a very short duration. In Japan, the seven most affected prefectures were Saitama, Chiba, Tokyo, Kanagawa, Osaka, Hyogo, and Fukuoka. Japan announced a state of national emergency and these counties were defined as emergency areas, while the rest of the country was defined as a non-emergency area. The general public suffered psychological distress during the COVID-19 pandemic (Sasaki et al., 2020; Shigemura et al., 2020). Therefore, we assessed the factors related to anxiety and the associated responses to the COVID-19 pandemic. These data may be used to develop and implement the emotional regulation control strategies in Japan.

The previous studies suggested that anxiety may cause psychological and behavioral changes, but few studies have assessed the associated reactions to anxiety, especially during a pandemic. In this study, we used a six-item state version of the State-Trait Anxiety Inventory (STAI-6) – a self-report psychological test for adults designed to measure feelings of immediate anxiety and the level of anxiety (Sydeman, 2018). We aimed to investigate the anxiety symptoms in the general public during the COVID-19 pandemic in Japan and explore reactions related to anxiety. We explored the relationship between STAI-6 Score, sources of COVID-19-related information, the influences of COVID-19, social anxiety symptoms, discrimination, and evaluation of the government.

## Materials and Methods

### Study Design

A cross-sectional web-based survey was adopted *via* an online market research company, Cross Marketing Inc. Japan^1^ (Shiina et al., 2021) between May 12 and 13, 2020 in Japan. The survey included a pool of approximately 2 million (the total population of Japan is 127 million) registered individuals. We used quota sampling to attain an equal gender and age distribution representative of the Japanese population, based on statistics from the Labor Force Survey and Ministry of Internal Affairs and Communications. The monetary incentive was provided to participants who completed the survey. We sent the survey link to 5,610 individuals and a total of 4,134 participants responded to the questionnaire. After data cleaning (the respondents with logically inconsistent answers were excluded), 4,127 complete responses were analyzed in this study (Cai et al., 2021).

### Questionnaire Design

This study consisted of questions on: (1) demographic characteristics (such as, age, gender, education, occupation, income, location, and with or without underlying disease); (2) individual anxiety; (3) sources of information on COVID-19-related news; (4) influences on individuals or their family from the COVID-19 pandemic; (5) social anxiety related to COVID-19; (6) discrimination related to COVID-19; and (7) evaluation score regarding the preventive activities undertaken by the national and local governments.

To assess individual anxiety levels related to the COVID-19 pandemic, the STAI-6 was used (Knight et al., 1983). This scale contains six items and four choices (1 = not at all, 2 = somewhat, 3 = moderately, and 4 = very much) that reflect common symptoms of anxiety experienced by an individual. The scores on the three positively worded items were reverse-coded, a higher score corresponded to a higher level of individual anxiety. STAI-6 is a simplified version of STAI-20, and the total summed scores were prorated (multiplied by 20/6) to obtain scores that were comparable to those from the full 20-item STAI (Knight et al., 1983; Marteau and Bekker, 1992).

The question on COVID-19 information sources was multiple choice and included TV, radio, government authorities, internet or social network service (SNS), newspaper, family, or friends. We used a self-report method to explore the influences of COVID-19 (increase in family quarrels and conflicts; weakening of social connections; increased intake of alcohol; increased smoking; increase in sexual harassment or violence; sleep quality affected; becoming nervous toward people around you; people around you became nervous and anxious; gained weight). Participants could choose more than one answer in the last item (included the impact on individuals and families).

The section on social anxiety consisted of five items that included fear, avoidance, keeping secrets, embarrassment, and stigma. The acts of discrimination contained feelings of not wanting to be in contact with anyone related to COVID-19, even though they were not contagious. There were four answer options in the section on social anxiety and discrimination in the original questionnaire, and we simplified the answers to “Yes” (Strongly agree and Agree) and “No” (Strongly disagree and Disagree). A scoring system was adopted to evaluate the preventive measures taken by the national and local governments. The scores ranged from 0 to 100, with higher scores indicating a higher evaluation of the government. All the variable values in our study are shown in Table 1.

**TABLE 1:** Variable value of constant in our study.

Constant	Variable value
Anxiety (STAI-6) score	Continuous variable
COVID-19-related social anxiety (Fear; Avoidance; Keeping COVID-19 a secret; Embarrassment; Stigma)	0 = No; 1 = Yes
Discriminatory behaviors (I don’t want to contact any COVID-19 positive person even though wearing PPE/I don’t want to contact any relative of COVID-19 positive person, even though he/she is tested negative/I don’t want to contact any health care worker who treated COVID-19 patients/All of the COVID-19 positive person should be isolated even though he/she is tested negative now	0 = No; 1 = Yes
Age	1 = 20–30; 2 = 31–40; 3 = 41–50; 4 = 51–60; and 5 = 61–70
Gender	1 = Male; 2 = Female
Highest educational level	1 = Primary school and below; 2 = Secondary and senior high school; and 3 = Junior college or vocational school and above
Occupation	1 = Informal occupation; 2 = Formal occupation; and 3 = Unemployed and others
Annually household income (10,000 JPY)	1 = ≤200; 2 = (200, 500]; 3 = (500, 800]; and 4 = >800
History of chronic disease	0 = No; 1 = Yes
Location	0 = No emergency area; 1 = Emergency area
Evaluate score of the preventive activities by the national government	Continuous variable
Evaluate score of the preventive activities by the local government	Continuous variable
Numbers of resources to receive COVID-19 related news	1 = ≤2; 2 = >2
Numbers of influences in individual or family caused by COVID-19	1 = ≤1; 2 = >1

### Ethical Considerations

This study was conducted with the approval of the Research Ethics Committee of Nagasaki Prefectural Institute of Environment and Public Health (No. 2020-6-1). Participants were informed that their participation was voluntary and they had the option to withdraw from the survey at any time before their participation. We used an online consent which was in accordance with the Declaration of Helsinki and approved by the ethics committee to obtain the informed consent of participants. The data are completely anonymous.

### Data Analysis

Means, frequencies, and SDs were used to quantify the data. We use Student’s *t*-test, *F*-test, and rank-sum test to identify differences in STAI-6 score in individuals with different demographic backgrounds. The sources of COVID-19 related information, social anxiety and discrimination related to COVID-19 were correlated to the STAI-6 score using the Spearman’s correlation test. We used a generalized additive model to analyze the factors of anxiety and evaluate the score of preventive activities by the national and local governments and their correlation with the STAI-6 score. Chi-square (χ*^2^*) was used to identify the relationship between individual anxiety and influences on individuals or their families. All the analyses also contained subgroup analyses stratified by type of emergency region. Data were analyzed using R software version 4.0.2 for Windows 10. Excel 2019 was used to draw figures.

## Results

### Demographic Characteristics and State-Trait Anxiety Inventory Score

There were 2,187 respondents from emergency areas and 1,940 respondents from non-emergency areas. The STAI score was range 6–24 (95% *CI* = 17.461–17.695). The differences in the mean STAI-6 scores were statistically significant regarding age, gender, highest educational level, occupation, income, underlying disease, and location (*p* < 0.05). Participants [annual household income was 200–500 (10,000 JPY)] from non-emergency areas had a higher mean STAI-6 score compared to participants in emergency areas (after Bonferroni correction). The detailed characteristics and STAI-6 scores of the participants are presented in Table 2.

**TABLE 2:** Demographic characteristics of participants and State-Trait Anxiety Inventory (STAI) score (*N* = 4,127).

Characteristics	*N* (%)	Location		| *t*| /| *z*|	*p*_1_	*F*/| *t*| /| χ^2^|	*p*_2_
		
		Emergency area (*N* = 2,187)	Non-emergency area (*N* = 1,940)				
		STAI-6 score (mean ± standard deviation)	STAI-6 score (mean ± standard deviation)				
Age (years)				*Bonferroni correction* α = 0.005		4.540	**0.001**
20–30	676 (16.4)	17.0 ± 0.2	17.3 ± 0.2	1.275	0.203		
31–40	802 (19.4)	17.3 ± 0.2	17.5 ± 0.2	0.645	0.519		
41–50	986 (23.8)	17.5 ± 0.2	17.8 ± 0.2	1.101	0.271		
51–60	825 (20.0)	17.8 ± 0.2	18.1 ± 0.2	1.183	0.237		
61–70	845 (20.4)	17.6 ± 0.2	17.8 ± 0.2	0.877	0.381		
Gender				*Bonferroni correction* α = 0.05		9.646	**<0.001**
Male	2,049 (49.6)	16.8 ± 0.1	17.2 ± 0.1	2.185	0.029		
Female	2 085 (50.4)	18.1 ± 0.1	18.2 ± 0.1	1.085	0.278		
Highest educational level				*Bonferroni correction* α = 0.017		4.220	**0.015**
Primary school and below	10 (0.2)	15.8 ± 2.6	19.2 ± 1.8	1.061	0.288		
Secondary and senior high school	1,227 (29.7)	17.8 ± 0.2	17.9 ± 0.1	0.188	0.851		
Junior college or vocational school and above	2,897 (70.1)	17.3 ± 0.1	17.6 ± 0.1	2.280	0.022		
Occupation				*Bonferroni correction* α = 0.017		7.942	**0.019**
Informal occupation	1,618 (39.1)	17.8 ± 0.1	18.1 ± 0.1	2.053	0.040		
Formal occupation	1,885 (45.6)	17.1 ± 0.1	17.3 ± 0.1	1.072	0.284		
Unemployed and others	631 (16.3)	17.6 ± 0.2	17.8 ± 0.2	0.663	0.508		
Annually household income (10,000 JPY)				*Bonferroni correction* α = 0.008		14.890	**<0.001**
≤200	678 (16.4)	17.8 ± 0.2	18.2 ± 0.2	1.463	0.144		
(200,500]	1,660 (40.2)	17.3 ± 0.1	17.9 ± 0.1	2.903	**0.004**		
(500,800]	1,056 (25.5)	17.5 ± 0.2	17.3 ± 0.2	0.719	0.472		
>800	740 (17.9)	17.4 ± 0.2	17.4 ± 0.2	0.106	0.916		
History of underlying disease				*Bonferroni correction* α = 0.05		7.391	**0.005**
Yes	1,383 (33.4)	17.1 ± 0.1	17.5 ± 0.1	2.366	0.018		
No	2,751 (66.6)	18.2 ± 0.1	18.2 ± 0.1	0.118	0.906		
Location						2.293	**0.022**
Non-emergency area	1,940 (47.0)	–	–	–	–		
Emergency area	2,187 (53.0)	–	–	–	–		

*T-test was used to compare the STAI-6 score between two group, multiple comparisons and homogeneity of variance were performed using F test and multiple group comparisons with uneven variances using rank sum test.*

*Bold values means P < 0.05.*

### Sources of Information and State-Trait Anxiety Inventory Score

**FIGURE 1:**
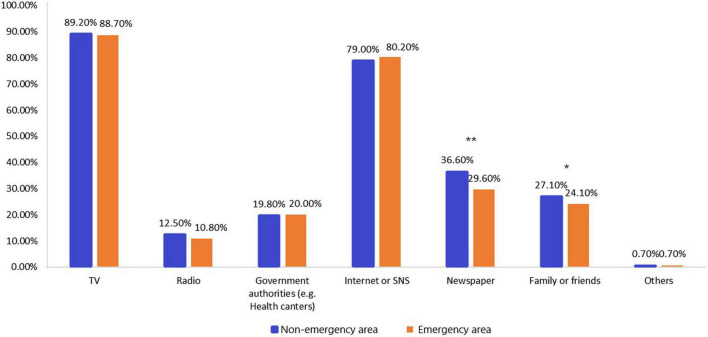
Sources of information for participants in different locations to receive coronavirus disease 2019 (COVID-19)-related news. *P < 0.05; **P < 0.01.

**FIGURE 2:**
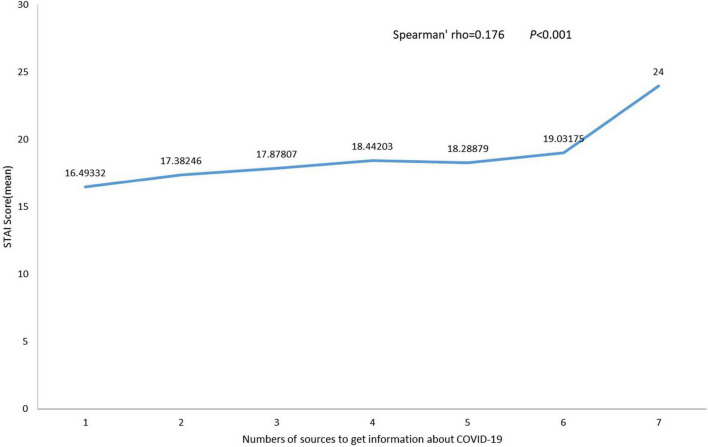
The relationship of State-Trait Anxiety Inventory-6 (STAI-6) score and numbers of sources to get information about the COVID-19.

The differences in sources of COVID-19-related news in different locations are shown in Figure 1. The number of participants from non-emergency areas that got information related to COVID-19 from the Newspaper (*p* < 0.01) and family or friends (*p* < 0.05) was larger than respondents from emergency areas. The number of sources of information on COVID-19 was positively correlated with the STAI-6 score (Spearman’s rho = 0.176, *p* < 0.001; Figure 2). Different locations also showed the same result (emergency area: Spearman’s rho = 0.166, *p* < 0.001; non-emergency area: Spearman’s rho = 0.186, *p* < 0.001).

### Influences of Coronavirus Disease 2019 and Individual Anxiety

**FIGURE 3:**
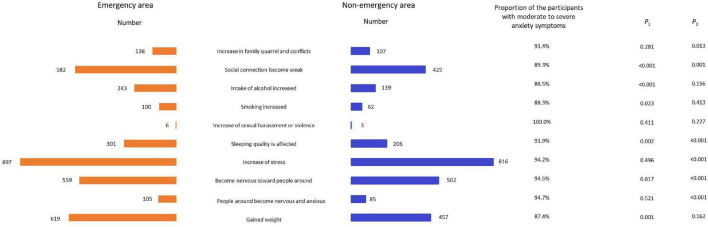
The situation of COVID-19 epidemic influence the participants or their family and the relation of moderate to severe anxiety symptoms. P1 means the significance of the differences in COVID-19 epidemic influences between different location; P2 means the significance of the differences in the incidence of moderate to severe anxiety symptoms amongst participants with or without COVID-19 epidemic influences (The influences included increase in family quarrels and conflicts; weakening of social connections; increased intake of alcohol; increased smoking; increase in sexual harassment or violence; sleep quality affected; becoming nervous toward people around you; people around you became nervous and anxious; gained weight).

Figure 3 shows the differences in COVID-19 pandemic influences between different locations and the relationship of these influences on individual anxiety. Participants with influences, such as weakened social connections, increased alcohol intake, increased smoking, sleep quality affected, and gained weight were more frequently from emergency areas (*p* < 0.05). The respondents that experienced an increase in family quarrels and conflicts, weakened social connections, sleep quality affected, increase in stress, became nervous toward people around them, and people around me became nervous and anxious were more likely to indicate moderate to severe individual anxiety symptoms (*p* < 0.05).

### Social Anxiety, Discriminatory, and State-Trait Anxiety Inventory Score

**FIGURE 4:**
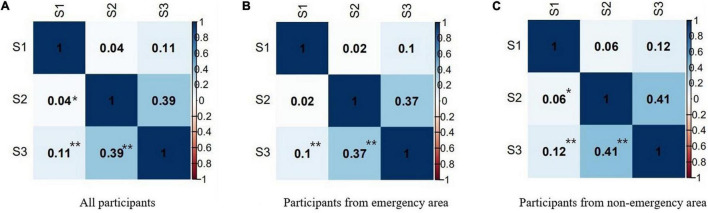
The relationship between STAI-6 score and numbers of social anxiety symptom and discriminatory behavior related to COVID-19. S1 = STAI-6 Score; S2 = Numbers of social anxiety symptom; S3 = Numbers of discriminatory behavior related to COVID-19. *P < 0.05; **P < 0.01.

The summary of social anxiety and discrimination related to COVID-19 in different locations is shown in Table 3. In all participants, there were positive correlations between the number of social anxiety symptoms, the number of discriminatory behaviors, and the STAI-6 score. Specifically, increased number of social anxiety symptoms and discriminatory behaviors were associated with increased STAI-6 scores. Participants from non-emergency areas had similar results. While in emergency areas, there was no correlation between the STAI-6 score and the number of social anxiety symptoms (Figure 4).

**TABLE 3:** Differences of social anxiety symptoms and discriminatory behaviors related to the coronavirus disease 2019 (COVID-19) between different locations.

Variables	*N* (%)	Non-emergency area (*N* = 1,940)	Emergency area (*N* = 2,187)	χ^2^	*P*
**Social anxiety symptoms**					
I will be afraid to let people know if I may have been infected with COVID-19				25.949	**<0.001**
Yes	2,709 (65.6)	1,351 (69.6)	1,358 (62.1)		
No	1,418 (34.4)	5,89 (30.4)	8,29 (37.9)		
If I suspect I may have been infected with COVID-19, I will not think about it until become unwell/sick				0.066	0.797
Yes	1,702 (41.2)	796 (41.0)	906 (41.4)		
No	2,425 (58.8)	1,144 (59.0)	1,281 (58.6)		
If I suspect I may have been in infected with COVID-19, I will keep it a secret				9.426	**0.002**
Yes	1,703 (41.3)	849 (43.8)	854 (39.1)		
No	2,424 (58.7)	1,091 (56.2)	1,333 (60.9)		
I will feel embarrassed if others know that I may have been infected with COVID-19				19.129	**<0.001**
Yes	1,807 (43.8)	919 (47.4)	888 (40.6)		
No	2,320 (56.2)	1,021 (52.6)	1,299 (59.4)		
I will lose friends if I tell them I may have been infected with COVID-19				11.561	**0.001**
Yes	1,054 (25.5)	543 (28.0)	511 (23.4)		
No	3,073 (74.5)	1,397 (72.0)	1,676 (76.6)		
**Discriminatory behaviors**					
I don’t want to contact any COVID-19 positive person even though wearing PPE				0.179	0.672
Yes	2,588 (62.7)	1,210 (62.4)	1,378 (63.0)		
No	1,539 (37.3)	730 (37.6)	809 (37.0)		
I don’t want to contact any relative of COVID-19 positive person, even though he/she is tested negative				6.071	**0.014**
Yes	2,359 (57.2)	1,148 (59.2)	1,211 (55.4)		
No	1,768 (42.8)	792 (40.8)	976 (44.6)		
I don’t want to contact any health care worker who treated COVID-19 patients				4.433	**0.035**
Yes	1,562 (37.8)	767 (39.5)	795 (36.3)		
No	2,565 (63.2)	1,173 (60.5)	1,392 (63.7)		
All of the COVID-19 positive person should be isolated even though he/she is tested negative now				0.247	0.619
Yes	3,057 (74.1)	1,444 (74.4)	1,613 (73.7)		
No	1,070 (25.9)	496 (25.6)	574 (26.3)		

*Bold values means P < 0.05.*

### Factors Associated With State-Trait Anxiety Inventory Score in the Generalized Additive Model

**FIGURE 5:**
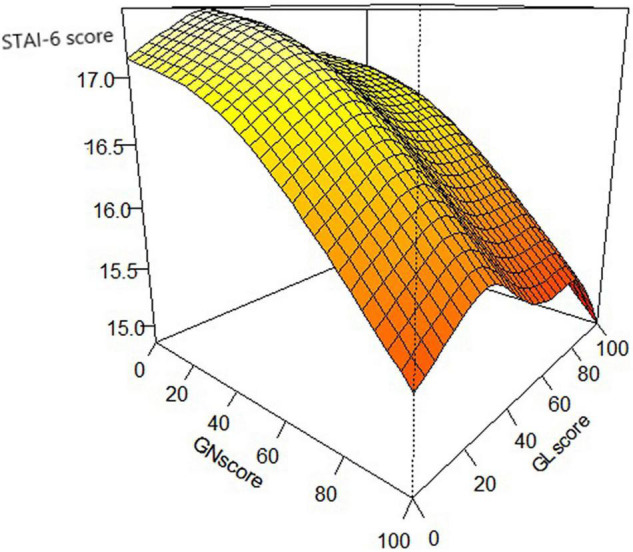
The evaluate score of governments affecting the STAI-6 score. GN Score = Evaluate score of the preventive activities by the national government; GL Score = Evaluate score of the preventive activities by the local government.

The generalized additive model included age, gender, education, occupation, income, location, number of resources for COVID-19 related news, number of influences on individual or family caused by COVID-19, an evaluative score of the preventive activities undertaken by the national and local governments as independent variables, and the STAI-6 score as a dependent variable. Being female (Estimate = 0.926) and from a non-emergency area (Estimate = −0.308) was related to higher STAI scores, and higher education (Estimate = −0.305) and income (Estimate = −0.168) decreased the STAI score. The respondents who had a lower evaluative score of the preventive activities undertaken by the national and local governments had higher STAI-6 scores (*p* < 0.05; Table 4 and Figure 5).

**TABLE 4:** The generalized additive model of STAI-6 score.

Parametric coefficients	Estimate	Std. Error	*t*	*P*
Intercept	13.788	0.495	27.846	**<0.001**
Age	0.055	0.043	1.296	0.195
Gender	0.926	0.118	7.823	**<0.001**
Education	–0.305	0.124	–2.462	**0.014**
Occupation	–0.075	0.085	–0.885	0.376
Income	–0.168	0.060	–2.777	**0.006**
Location	–0.308	0.113	–2.719	**0.006**
Numbers of resources to receive COVID-19 related news	0.692	0.116	5.976	**<0.001**
Numbers of influences in individual or family caused by COVID-19	1.904	0.124	15.345	**<0.001**

**Approximate significance of smooth terms**	**Estimated degree of freedom**	**Referenced degree of freedom**	***F***	***P***

Evaluate score of the preventive activities of the nation government	2.039	2.572	13.866	**<0.001**
Evaluate score of the preventive activities of the local government	4.203	5.148	2.201	**0.048**

*Bold values means P < 0.05.*

## Discussion

Our study provided insight into the reactions related to individual anxiety during the COVID-19 pandemic. Higher access to information regarding COVID-19 increased anxiety and influences related to COVID-19 added the possibility of moderate to severe anxiety symptoms. The increased level of anxiety may lead to social anxiety symptoms, discriminatory behaviors. And distrust in the government also related to anxiety. In addition, we suspect that a high level of anxiety may increase the possibility of being affected by the COVID-19 pandemic.

To date, numerous studies have explored anxiety in the general public, health workers, teachers, students, and patients with certain diseases during the ongoing COVID-19 pandemic. These studies can be broadly divided into three categories. The first category of risk factors (such as, sociodemographic factors, online social media information, and knowledge of COVID-19) related to anxiety, has been studied the most (Jo et al., 2020; Jungmann and Witthöft, 2020; Liu C. et al., 2020; Yang et al., 2021). The second type of study focused on the effects of interventions on anxiety caused by the COVID-19 pandemic (Chen, 2020; Liu K. et al., 2020; Wei et al., 2020); interventions mainly consist of relaxation techniques like breathing relaxation training and progressive muscle relaxation. The instructions for these interventions were audio-recorded and put online, and the researchers assessed anxiety before and after the intervention. Physical activity is also an intervention, and moderate physical activity during the COVID-19 pandemic has the proven benefit of resulting in a more positive mental state and decreased anxiety (Reigal et al., 2021). The third type focused on how anxiety influences daily life, and behavior changes caused by anxiety were common among the general public during the COVID-19 pandemic (Stanton et al., 2020). In addition, studies on anxiety related to work efficiency were mainly conducted in a certain type of worker (Sasaki et al., 2020), while investigations on patients in hospitals explored changes in somatic function due to anxiety (Speth et al., 2020). To our knowledge, there are few studies focused on well-known anxiety-associated reactions during the COVID-19 pandemic. Our findings provide a clear, serial response to anxiety and offer informative implications for addressing psychological problems during a pandemic.

In our investigation, anxiety was not equally distributed across the general population with respect to age, gender, education level, occupation, income, history of underlying disease, and location. These results were in accordance with a recent national investigation in Japan (Qian and Yahara, 2020).

During the pandemic, people were instructed to stay at home, particularly in emergency areas, and sources of COVID-19 related information played a central role in understanding the status of the pandemic. Our study found that the two highest sources of information were TV and the internet or SNS. The number of responders from non-emergency areas who got information from the newspaper and family or friends was higher in emergency areas. These findings are consistent with current precautionary measures taken by Japan.

Indeed, acquiring information from various sources can add to fear and generate anxiety (Jones and Salathé, 2009). In this study, the number of resources for COVID-19 information and anxiety level had a strong, positive correlation in both emergency and non-emergency areas, confirming the hypothesis that getting numerous messages about a pandemic increased anxiety. A report conducted in Russia found that excessive media consumption about COVID-19 was related to anxiety (Nekliudov et al., 2020), which confirms that consuming a large amount of relevant information can increase anxiety related to COVID-19.

Concerning the influence of the COVID-19 pandemic on individuals or their families, we found that the respondents from emergency areas were more likely to be affected by the pandemic. Therefore, we speculated that during the period that people had to stay at home, family quarrels and conflicts may have increased and social connections had become weaker. Due to the diminution of social intercourse, people became nervous toward others and thought that others were nervous too. They also felt that stress increased and sleep quality was affected. In addition, COVID-19 was related to anxiety symptoms, and our present study shows that the people who were more vulnerable to the effects of a pandemic were more likely to have moderate to severe individual anxiety. During the earlier severe acute respiratory syndrome (SARS) pandemic, researchers found that people experiencing the pandemic felt more stress (McAlonan et al., 2007) and fear (Leung et al., 2005), and were more prone to anxiety. The same reaction was found in our study of the COVID-19 pandemic and proves the feasibility of our research.

Social anxiety and discriminatory behaviors have been prevalent during this pandemic (Choi et al., 2020; Zheng et al., 2020). Our findings indicated that social anxiety symptoms and discriminatory behaviors may be the result of a high level of individual anxiety. Further, these factors may interact, meaning that social anxiety can increase discriminatory behaviors. A previous study on immigrants suggested that perceived discrimination increased social anxiety symptoms (Fang et al., 2016). Our study is the first to discuss individual discriminatory behaviors and social anxiety symptoms. The results showed that people who discriminated against COVID-19 positive people or relevant health workers suffered from more social anxiety symptoms. We used the generalized additive model to explore the relationship between trust in governments (national and local) and anxiety. Findings indicated that accounting for the effect of different regions, participants with a lower evaluation of the government’s preventive measures had a higher level of anxiety. These findings are consistent with other studies showing that trust and anxiety have a negative predictive relationship (Zwingmann et al., 2017; Pamela et al., 2019).

During an infectious disease pandemic, people may change and modify their behavior to adapt and achieve their needs (Reigal et al., 2020). Our investigation showed that impacts from the COVID-19 outbreak increased the likelihood of anxiety. We suspected that people with higher levels of anxiety were more influenced by the COVID-19 pandemic. Previous reports showed that individuals with high anxiety were more affected by disease or public health emergencies (Asmundson and Taylor, 2020; Yang et al., 2020), and that anxiety added to the possibility of being affected by a pandemic, which confirmed our hypothesis.

This study had some limitations. First, as this was an online investigation, participants who did not use the internet were not included, which may limit the generalizability of the results. The questionnaire was designed for the public, and questions for specific groups (such as those who tested positive for COVID-19) were not included. In addition, self-report questionnaires contained recall bias and may be influenced by social desirability biases. Moreover, it is difficult to infer causal relationships from cross-sectional studies. Thus, studies employing multiple follow-up assessments are needed to characterize anxiety and its related reactions. Lastly, the correlation effect was small in our study, which may be because psychological changes are not obvious, and prospective studies are needed to verify these findings. Despite the limitations, our internet-based sampling strategy was quickly deployed and could thereby track responses in near real-time and a large sample size with varied demographic backgrounds. Additionally, we used a standardized questionnaire (STAI-6) to diagnose anxiety, which supported the viability of the data. These findings provide insight into anxiety and related responses during a pandemic.

## Conclusion

The current study was the first to provide evidence for the association between anxiety and increased access to information on COVID-19. Additionally, the outbreak and distrust in the government added to anxiety, and a high level of anxiety increased social anxiety, discriminatory behaviors, while anxiety may increase the possibility of remaining under the influence of COVID-19. Our findings may aid psychologists by providing new knowledge on anxiety and serve as a critical step in developing a strategic plan to address the later dimensions of anxiety due to a pandemic. Furthermore, these findings may be generalized to other diseases or traumatic events.

## Data Availability Statement

The raw data supporting the conclusions of this article will be made available by the authors, without undue reservation.

## Ethics Statement

The studies involving human participants were reviewed and approved by the Research Ethics Committee of Nagasaki Prefectural Institute of Environment and Public Health (No. 2020-6-1). The participants provided their written informed consent to participate in the study.

## Author Contributions

GC and FH designed the study. JZ, GC, YLu, XX, and YLi wrote the original draft of the manuscript. YLu, YLi, and GC contributed to the investigation. JZ and FH analyzed the data. LW, ZH, TY, KM, and KA contributed to the interpretation of the results. XX modified the format. All authors reviewed the manuscript and approved the submitted version.

## Conflict of Interest

The authors declare that the research was conducted in the absence of any commercial or financial relationships that could be construed as a potential conflict of interest.

## Publisher’s Note

All claims expressed in this article are solely those of the authors and do not necessarily represent those of their affiliated organizations, or those of the publisher, the editors and the reviewers. Any product that may be evaluated in this article, or claim that may be made by its manufacturer, is not guaranteed or endorsed by the publisher.

## Footnotes

1
https://www.cross-m.co.jp/en/
